# Community Feedback on Mass Medicines Administration for Neglected Tropical Diseases in Federal Capital Territory, Abuja, Nigeria

**DOI:** 10.3390/tropicalmed9060126

**Published:** 2024-05-30

**Authors:** Juliana Ajuma Amanyi-Enegela, Jacqueline Azumi Badaki, Gbenga Olorunshola Alege, Faizah Okunade, Joseph Kumbur, Rinpan Ishaya, Donald Ashikeni, Mohammad Babar Qureshi, Girija Sankar

**Affiliations:** 1 Inclusive Eye Health and Neglected Tropical Diseases Initiative, CBM Christoffel-Blindenmission Christian Blind Mission e.V., Wellington House, East Road, Cambridge CB1 1BH, UK; 2 Department of Zoology, Federal University Lokoja, Lokoja 260101, Nigeria; 3 Department of Biotechnology, Federal University Lokoja, Lokoja 260101, Nigeria; 4 CBM Christoffel-Blindenmission Christian Blind Mission e.V Nigeria Country Office, 13 Okemesi Crescent, Garki 2, Federal Capital Territory, Abuja 900103, Nigeria; 5 HANDS, 5A Naomi Jugu Drive, Rayfield, Jos, Nigeriadonald.ashikeni@handsnigeria.org (D.A.)

**Keywords:** community engagement, community feedback, mass drug administration, neglected tropical diseases, Sub-Saharan Africa, Nigeria

## Abstract

The World Health Organization (WHO) recommends the use of annual mass drug administration (MDA) as the strategy for controlling and eliminating the five preventive chemotherapy neglected tropical diseases (PC-NTDs). The success of MDAs hinges on community acceptance, active participation, and compliance. This study aimed to explore the experiences and perceptions of community members, to obtain a more thorough understanding of their openness and willingness to participate in MDA and other NTD elimination activities. A mixed-methods approach was employed, utilizing qualitative and quantitative methods for comprehensive data collection. Eighteen key informant interviews (KIIs) and sixteen focus group discussions (FGDs) were conducted to explore community engagement, participation, medication utilization, and programme perception. Triangulation of findings from interviews and discussions with household survey results was performed to gain a deeper understanding of emerging themes. The household survey involved interviewing 1220 individuals (Abaji: 687; Bwari: 533). Audio tapes recorded KIIs and FGDs, with interview transcripts coded using Nvivo 12.0 software based on predefined themes. Descriptive analysis using SPSS version 21 was applied to quantitative data. Results indicated high awareness of mass drug administration (MDA) campaigns in both area councils (Abaji: 84.9%; Bwari: 82.9%), with a small percentage claiming ignorance (15.1%), attributed to lack of information or absence during health campaigns. Respondents primarily participated by taking medication (82.5%), with minimal involvement in other MDA campaigns. Perception of medicines was generally positive, with a significant association between participation level and performance rating (*p* < 0.05). The study recommends leveraging high awareness and community responsiveness to enhance engagement in various MDA activities, ensuring sustainability and ownership of the programme.

## 1. Introduction

Neglected tropical diseases (NTDs) remain a significant public health challenge globally, particularly in low- and middle-income countries where they disproportionately affect marginalized communities [1]. Nigeria, as one of the most populous countries in Africa, bears a substantial burden of NTDs, with prevalence rates varying across different regions [2]. The prevalence and impact of NTDs are significant in the Federal Capital Territory (FCT), Abuja. FCT is endemic for onchocerciasis, lymphatic filariasis, schistosomiasis, and soil-transmitted helminth infections [3].

The World Health Organization (WHO) recommends community-wide annual mass drug administration (MDA) as the strategy to interrupt the transmission and elimination of these four NTDs including trachoma [4]. MDA is implemented using a community-driven approach in which various stakeholders assume specific roles in a coordinated effort to ensure the success of the campaigns. Locally, community members identify volunteers through methods determined by their communities. These volunteers are trained to distribute PC-NTDs medicines. Community leaders and opinion influencers are also tasked with disseminating information on drug availability and the importance of adhering to annual treatments. MDA campaigns have been pivotal in reducing the morbidity and mortality associated with NTDs by providing preventive chemotherapy to at-risk populations [5].

With over two decades of NTD control and elimination activities in FCT, community fatigue may delay reaching the final elimination mile. Additionally, communities are also evolving due to global influences. Given the dynamic context in which NTD campaigns operate, community feedback is an invaluable tool to assess the effectiveness of public health interventions such as NTD programmes, ensuring they resonate with the targeted communities and remain relevant to local realities [6]. Communities are essential for the success of NTD programmes. Therefore, understanding community perspectives on MDAs and identifying their challenges can provide valuable insights into factors affecting their participation. This knowledge can then be used to enhance the effectiveness and sustainability of NTD control programmes.

This study explored community members’ experiences and perceptions of mass drug administration campaigns to inform improved implementation strategies and enhance community health outcomes.

## 2. Methods

### 2.1. Study Design

A mixed-methods, cross-sectional research design comprising surveys, key informant interviews, and focus group discussions (FGDs) was used to explore community engagement, participation in mass drug administration, and perceptions of NTD campaigns. Qualitative methods included key informant interviews (KIIs) and focus group discussions (FGDs), while quantitative data were gathered through household surveys.

### 2.2. Description of Study Area

The study was conducted in the Federal Capital Territory (FCT), located centrally in Nigeria with an area of approximately 7315 square kilometres. FCT is bordered by Niger State to the West and North, Kaduna State to the Northeast, Nasarawa State to the East and South, and Kogi State to the Southwest. It has an estimated population of 3.4 million as of 2021 and consists of six area councils. The study specifically targeted Abaji and Bwari area councils within the FCT.

### 2.3. Selection of Study Sites

**Figure 1:**
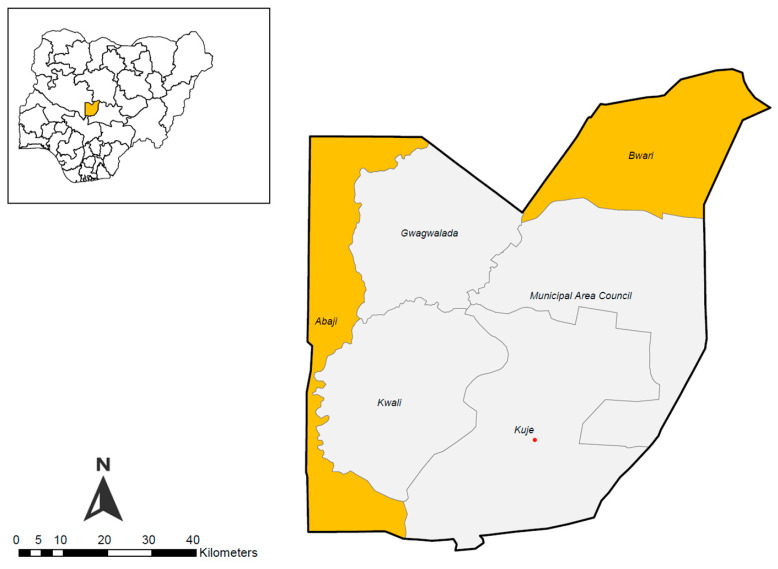
Map of Nigeria (upper left) with FCT highlighted in yellow and the map of FCT with study areas highlighted in yellow.

The Federal Capital Territory (FCT) has six area councils that are also endemic for at least two of the five PC-NTDs (onchocerciasis, lymphatic filariasis, schistosomiasis, and soil-transmitted helminthiasis and trachoma) earmarked for elimination by WHO. Thus, MDA campaigns have been ongoing in FCT for almost two decades. Abaji and Bwari area councils were selected as they were among the areas where NTD control and elimination activities commenced in FCT. Additionally, both area councils have reported dwindling treatment coverages for the endemic NTDs over the past three years, as indicated by trend analyses of reported coverage. The map of the Federal Capital Territory (FCT) in Nigeria in Figure 1 shows the study areas highlighted in yellow.

### 2.4. Sampling

The primary sampling unit comprised communities within both area councils. The sampling frame included 89 communities for Abaji and 197 communities for Bwari area councils. Ten communities were selected per council using the sample function in R software: version 4.1.2 [7]. The secondary sampling units were Households. For the survey, households were chosen by randomly selecting a quadrant and employing a fixed ^n^p00ppppsampling interval where 12 households were selected using a spin of a bottle. Assuming a minimum household size of 5, we aimed to interview 60 respondents per community. Nevertheless, in cases where communities had very small household sizes, the number of households visited was increased to compensate for the shortfall.

In each area council, we assumed a 70% utilization of NTD services, denoted as *p* = 0.7 [*p*], with a 95% confidence level corresponding to a z score of 1.96 [Z], a degree of accuracy of 0.05, and a non-response rate of 10%. Consequently, a minimum sample size of 215 individuals per area council was calculated using an online sample size calculator: http://www.surveysystem.com/sscalc.htm (accessed on 28 August 2022). A total of 1220 individuals were included in the household survey.

### 2.5. Data Collection

A combination of quantitative and qualitative methods was used for data collection, which commenced in September and continued through December 2022. Six research assistants who satisfied the minimum criterion of local language proficiency were recruited and trained to conduct the survey, KIIs, and FGDs.

One thousand two hundred and twenty individuals (Abaji: 687; Bwari: 533) were interviewed in the household survey while eighteen (18) key informant interviews (KIIs) and sixteen (16) focus group discussions (FGDs) were used to gain insight on community engagement, participation, utilization of MDA and perception of the NTD programmes.

The survey was administered using the Kobo Collect app on Android smartphones, which were pre-loaded with the survey forms.

Key informant interviews and focus group discussions were recorded using encrypted audio tapes and field notes. The KIIs were conducted with community leaders, opinion leaders, and community drug distributors (CDDs). Participants of FGDs were male and female members of communities of various age groups. Adult discussion groups were held separately from the youth groups. The findings from the interviews and discussions were triangulated with findings from household surveys for an in-depth comprehension of emerging themes. The data collected are summarized in Table 1.

### 2.6. Data Quality Control

To ensure data quality, a three-day training was conducted to equip research assistants with the required skills to conduct and document interviews and facilitate FGDs. Supervision was maintained throughout the data collection process.

### 2.7. Data Analysis

Interview transcripts were coded using Nvivo 12.0 software based on pre-defined themes. Quantitative data were analysed using SPSS version 21 to generate frequencies, and chi-square tests were used to compare frequencies, with a significance level set at *p* < 0.05. Triangulation of findings from qualitative and quantitative data sources enhanced the depth of understanding of emerging themes (MDA awareness, community participation, perceived benefits of NTD drugs, cultural beliefs, and CDD workload).

## 3. Findings

Our findings are presented and discussed under themes that could shed insight on the perspectives of community members regarding the various MDA campaigns for NTDs.

### 3.1. Mass Drug Administration (MDA) Awareness

MDA campaigns have been going on in the study area for over twenty (20) years, so awareness about the MDA campaigns for the different NTDs was high (Abaji (84.9%) and Bwari 82.9%) in both area councils (Table 2).

Amongst the 16.0% who responded as being unaware of any of the NTD programmes, the majority mentioned lack of information about MDA campaigns as a reason for unawareness. Other respondents (Abaji: 20.2%, Bwari: 21.2%) admitted that they were not fully settled in the community because they may have travelled and therefore could have missed out on any information regarding NTD programmes. There was no statistical significance in the responses provided on awareness and reasons for unawareness in both area councils.

Similarly, a KII respondent (community leader) from Tudun Fulani community hailed the high awareness as narrated below:

*“Health workers came over to the palace and we discussed the selection of teachers and CDDs with them. We selected two (2) teachers and they worked with the health workers. Fortunately, I used that opportunity to include the Fulanis (nomads), because we have some Fulani teachers amongst the people that participated in the training. They came over here, collected the drugs, and distributed them, ensuring that all Fulanis in their settlements were reached”.*

### 3.2. Community Participation

Participation in the NTD campaigns involved attending community meetings, holding pre-implementation discussions, awareness creation and health education about NTDs including informing communities about the availability of medicines, house-to-house distribution of the medicines by CDDs, and swallowing of medicines by community members.

Drawing from respondents’ awareness of the programme, their participation in specific NTD interventions (Oncho/LF and Schisto/STH) is reported in Table 3.

Table 3 shows that only one-third (36%) of the respondents from the Household Survey (HHS) participated in at least one component of the Oncho/LF MDA campaigns, while over 55% of respondents reported that their children swallowed the drug (praziquantel) for Schisto/STH. Responses from Abaji showed over 70% participation during Schisto/STH MDA campaigns, while Bwari reported 35% participation. In contrast, 64% of the interviewed individuals in Bwari participated in Oncho/LF MDA campaigns with just a 14.7% level of participation in Abaji. This is probably due to the scale-down of LF treatment in 2020 with Oncho MDA focused on only rural areas.

Regarding participation in different components of MDA campaigns, Table 4 shows that most of the respondents in Abaji (84.7%) and Bwari (83.1%) participated in swallowing the MDA medicines—an indication of high treatment coverage. There were also respondents in Abaji (5.7%) and Bwari (3.8%) who engaged in disseminating information about the availability of medicines to community members. Few respondents from Abaji (2.3%) and Bwari (2.8%) reported attending meetings regarding MDA campaigns while 4.8%, 1.6%, and 2.5% participated in disseminating information about the NTD medicines, engaged in providing health education regarding the NTDs and attended community meetings where MDA campaigns were planned, respectively. Table 4 provides a breakdown of responses by area council:

Participants in the distribution of the drugs were similarly few (Abaji:1.9%, Bwari: 1.7%)), but this is understandable since drug distribution is within the purview of CDDs. Community meetings were also typically attended only by heads of households because of the religious inhibitions which do not allow women to attend community-wide meetings with males.

Remarks from KIIs with community leaders and CDDs supported the above findings. One of the excerpts from the KII with a CDD in Kawu reads that:

*Many of my people came out to collect and use these medicines when we started distribution. Other community members took it upon themselves to move around the village to inform them about the MDA campaigns.*

(CDD, Kawu community, 2021)

Interestingly, the majority of those who did not participate in the NTD programme were willing to participate in future MDA campaigns because the reason for non-participation was not an aversion to the drug but absence from the community at the time medicines were distributed. As summarised in Table 5, 99% and 97.9% consented to participate in future NTD campaigns in Abaji and Bwari, respectively.

The absence of a health centre and frontline health workers to coordinate NTD activities in a community were reported as key factors that could hinder participation. The response below by a CDD (KII) underscores why the absence or distance of a health centre may affect access to essential life-saving health services:

*For example, in Tudun Fulani community, the nearest PHC is in Kawu Nomadic which is about two hours’ drive. The distance also comes along with the cost of transport to the PHC to access care. Their poor socio-economic status renders the situation difficult and almost impossible to use the PHC.*

### 3.3. Community Perceptions

A perceived benefit of MDA campaigns among respondents was improved health status as observed from the household survey and KII responses. This further explains the high proportion of respondents who ingested the NTD drugs.

The perceived benefits derived from Mectizan and Albendazole followed the same trend in both area councils. Most respondents reported a feeling of well-being after taking Mectizan and Albendazole—76.6% and 77.7% of them stated that they “felt better” after taking the drug in Abaji and Bwari area councils, respectively. Very few respondents in Abaji (1.0%) and Bwari (1.1%) LGAs, respectively, reported no visible change in health. More respondents in Abaji area council (7.3%) reported adverse effects ranging from vomiting to diarrhoea.

Similarly, in Abaji, 78.7% of respondents reported feeling better after taking praziquantel (Table 6). More than half (65.7%) of the respondents in Bwari reported that their children did not take the drug as SCH MDAs campaigns commenced in this study area only in 2019 and were fully scaled up in 2021.

Less than 1% claimed they felt no improvement in health status after taking either of the NTD drugs (Table 6). Adverse effects were very minimal: 3.9% and 3.2% of individuals reported experiencing side effects after swallowing Mectizan/albendazole and praziquantel, respectively.

*“People are complaining that the number of tablets they swallow overpowers them but at last they are appreciative because of what we see when we pass stool”.*

(KII with a Community Leader in Tudun Fulani Community, Bwari Area Council, 2021)

Regarding severe adverse events (SAE) of MDA medicines, one FGD participant recounted fear due to SAE of praziquantel below:

*“I was very scared, my daughter and grandchildren got really sick after swallowing praziquantel, and they were stooling and vomiting a lot. However, they recovered in no time and are feeling stronger than before taking the medicine”.*

(Community Member, HHS, Angwan Manko, Abaji)

Cultural beliefs play a significant role in how communities respond to public health programmes. Male participants in the FGDs shared that cultural norms, customs, and beliefs could dissuade people from participating in the programme. Culture, in their view, connotes a way of life, and it is transmitted from one generation to another. In this regard, the introduction of MDA in eliminating NTDs such as lymphatic filariasis contrasts with people’s beliefs about what they conceive as the cause and, therefore, the means of treating it. For example, one participant buttressed his point in this manner:

*“While growing as a child, we have been told that having big legs could only be induced by an evil spirit as punishment for breaking a taboo or as a victim of witchcraft, and in such case, spiritual approach is the remedy. We are here because of the awareness we have, but many still believe in these myths”.*

(Male FGD, Kawu, 2021)

Related to the above point is the stigmatization of people living with NTDs. In another informal discussion, one of the participants shared that stigma could be one of the reasons for lower participation in NTD campaigns. According to the participant:

*Many who have elephantiasis are called some kind of derogatory names that are demeaning and could affect one’s mental reasoning such that they become ashamed to seek treatment when the programme is ongoing. The fear of being mocked could deter such patients from participating in the programme.*

(Male FGD, Zango, 2021)

### 3.4. CDD Work Load and Incentive

Respondents were of the view that the government should remunerate CDDs to motivate them and ensure retention within the programme. This sentiment was captured in the following quotes from respondents across communities:

*I have been a CDD for many years and the job of distributing the NTD medicines is difficult. I am aware of some CDDs who have left due to poor incentives for such a demanding job. I think more people should be recruited as CDDs.*

(KII, CDD, Usafa, Community, Bwari Area Council)

*I am the only CDD for the Fulani community here and our population is about 7000. The process of house-to-house distribution of NTD medicines during MDA is demanding, however, I engage in the services for the love I have for my people.*

(KII, CDD. Tudun Fulani, Community-Bwari Area Council)

CDDs reported that motivation and compensation are areas that need to be addressed to improve retention during NTD MDA campaigns. The response from a KII with a CDD highlights the ongoing discussion about incentives in his community:

*“Some CDDs are dissatisfied with the incentive and poor transportation cost provided to us during training. They are discouraged and complaining that the money is too small”.*

(KII, CDD, Abaji Area Council)

### 3.5. Rating of MDA Campaigns

Respondents were requested to rate the performance of the programmes qualitatively and the ratings are summarised in Table 7. More than half of the respondents in both area councils (Bwari: 54.6%; Abaji: 50.1%) rated the MDA programme for Oncho/LF as excellent (Table 7). None of the respondents in Abaji rated the Oncho/LF programme “poor”, an indication that they are satisfied with the programme despite the drawback of CDD motivation. Respondents in Abaji also seemed to have high confidence in the Schistosomiasis/Soil-transmitted helminths programme because 70.5% rated the programme as performing excellently. About 54.6% of respondents in Bwari rated Schistos/STH as excellent, while 23.6% rated the programme as having performed “poorly” (Table 7).

In Abaji, about 12.2% and 14.0% assented that the Oncho/LF and Schisto/STH MDA campaigns had performed moderately well, respectively. The high confidence respondents in Abaji have in the Schisto/STH programme as compared to Bwari may be due to the perceived benefits of the medicine as reported previously in Table 2. There was also a strong positive association (*p* < 0.05) between participation and rating of the programme in both area councils (Abaji: χ_2_ = 465.75; Bwari: χ_2_ = 4649.66).

A majority (98.5%) of all the respondents from HHS were willing to participate more in NTD campaigns. In Abaji, 99% assented to participate in future NTD campaigns and 97.9% of the respondents in Bwari had a similar response (Table 6).

## 4. Discussion

In this study, we investigated community perspectives on mass drug administration (MDA) campaigns for neglected tropical diseases (NTDs) in the context of Abaji and Bwari area councils in FCT, Nigeria. Our findings provide valuable insights into the challenges and factors influencing community participation in MDA activities, shedding light on areas for improvement in NTD elimination programmes. By exploring themes such as MDA awareness, community participation, perceived benefits of NTD drugs, cultural beliefs, and the role of community drug distributors (CDDs), the findings of this study contribute to the existing knowledge on MDA programmes and offer recommendations for enhancing their effectiveness and sustainability.

### 4.1. High Awareness and Community Participation

The high awareness levels observed in both the Abaji and Bwari areas indicate the effectiveness of long-standing MDA campaigns, with over 80% of respondents being aware of the NTD elimination programmes. MDA awareness is a critical determinant of the extent of people’s involvement in a community-based campaign because information and knowledge could impact how communities choose to participate in a programme. This is consistent with a similar study in Liberia [8], which showed that limited awareness of diseases and the associated interventions influenced the demand for and acceptance of MDA in communities. This high awareness can also be attributed to various factors, including sustained engagement with community leaders, involvement of local teachers, and targeted outreach efforts, as highlighted by key informant interviews (KII) and focus group discussions (FGDs). This is consistent with findings of studies conducted in India [9] and Philippines [10] which suggest that regular community engagement increased awareness and community members’ willingness to participate in MDA campaigns.

Given the high awareness and usage of NTD drugs in the study area, one might expect robust participation in other components of MDA campaigns, such as planning, health education, and community meetings. However, involvement in these aspects was limited, with only a small portion of community members actively participating. Similarly, the number of individuals engaged in drug distribution was low, understandably as this responsibility falls within the purview of CDDs.

Cultural constraints restricted the attendance of women at community meetings, further constraining participation. Nonetheless, other community members supported awareness initiatives about MDA. Empowering these individuals alongside CDDs to become change agents in their communities could facilitate positive change within communities, fostering greater engagement in MDA activities. Women could potentially have a great impact on the success of MDAs as they exert significant influence on the health of children. The informal discussions that women hold are very powerful drivers of social change in communities [11]. Moreover, enhanced community involvement holds promise for improving health outcomes, service accessibility, and responsiveness, as noted by previous studies [12,13]. Additionally, the nature of information conveyed to community members may influence their level of participation in MDA activities. Findings from studies conducted by Kisoka, Njomo and colleagues indicates that insufficient information can impact community members’ choices regarding participation in MDA initiatives [14,15].

### 4.2. Community Perception and Cultural Norms

Community perceptions, experiences, and understanding of NTDs and the benefits of MDA medicines are critical factors that influence coverage rates and compliance with prevention measures. The positive perception of MDA programmes among respondents, reflected in the majority reporting improved health after drug ingestion, aligns with the intended goals of these interventions [5]. Results from this study indicated that community members perceived health improvements following ivermectin intake, despite some reporting prior adverse events; they persisted in taking the provided MDA drugs, aligning with the study by Vanamail and colleagues [16] in Southern India showing compliance with six rounds of ivermectin treatment despite initial adverse reactions. Insights from community members regarding the tangible benefits of the drugs, such as improved health outcomes, further emphasize the importance of sustained MDA efforts.

Cultural norms play a significant role in shaping beliefs, behaviours, and healthcare-seeking patterns within communities. Recognizing and understanding these norms is crucial as it enables the design of tailored interventions that can effectively improve health outcomes [17]. One notable challenge identified in achieving the last mile elimination goals is the community’s perception of lymphatic filariasis (LF)-related morbidity as a divine punishment, leading to social exclusion and stigma against affected individuals. In a study in Indonesia, respondents suggest that traditional medicine protects them from LF, thus excluding themselves from participating in MDA campaigns [18]. Such misconceptions about the benefits of LF MDA, cause of LF and its associated morbidity have the potential to hinder self-reporting and acceptance of treatment, thus posing a barrier to achieving LF elimination goals [19].

### 4.3. Community Drug Distributors (CDDs) and Retention

Community drug distributors (CDDs) are community-selected volunteers tasked with drug distribution, but their responsibilities extend far beyond this role. They play a crucial part in sensitizing and mobilizing communities for MDA campaigns, conducting censuses, providing health education, and maintaining treatment records for all NTDs. However, both community leaders and CDDs frequently cite the heavy workload of CDDs and its adverse effects, a concern highlighted in previous studies [20,21]. The lack of sufficient remuneration for CDDs during MDA campaigns emerges as a significant issue, leading to high attrition rates among CDDs and resulting in a few individuals being responsible for treating large populations [22].

While it is recognized that CDD roles are voluntary, the issue of CDD motivation continues to challenge NTD programmes [21,23]. Studies indicate that volunteerism without adequate motivation is not sustainable [24]. In our study, CDDs expressed dissatisfaction with the incentives they received, stating how this contributes to attrition and advocated for monetary benefits. The attrition of CDDs has been associated with insufficient financial incentives and the availability of other employment opportunities. In 2015, da-Costa Vroom and colleagues observed that CDDs generally earned less because the MDA period frequently coincided with the lucrative cocoa harvesting and small-scale gold mining seasons [25]. Rewards have the potential to bolster CDD morale, foster a high level of commitment to MDA services, and enhance overall satisfaction among CDDs.

A likely reason for the high level of willingness and resilience of CDDs could be adduced to the perceived benefits from the MDAs and improved relationships with community members [26].

### 4.4. Programme Performance and Future Participation

Overall, the favourable ratings of MDA campaigns underscore community satisfaction with programme performance, particularly in Abaji, where no respondents rated any programme as “poor”. The strong willingness expressed by respondents to participate in future campaigns signifies a positive outlook and highlights the importance of continued community engagement and support for sustainable NTD elimination efforts.

## 5. Conclusions

Our study highlights the complex dynamics surrounding community perspectives on MDA campaigns for NTDs. Addressing challenges related to equitable participation, access to hard-to-reach communities, cultural beliefs, and support for CDDs is crucial to enhance the effectiveness and sustainability of these interventions. Programme implementers should capitalize on the positive aspects identified in our findings to enhance community engagement in future MDA campaigns. This may involve increasing the involvement of community members in health education, ensuring the representation of both male and female community leaders in decision-making meetings, and fostering sustainability and ownership of interventions. Recommendations include targeted community outreach, gender-sensitive and culturally appropriate approaches to address misconceptions, increasing access for communities without health centres, strengthening support mechanisms for CDDs, and continuous monitoring and evaluation to optimize outcomes and ensure community participation and ownership.

### Study Limitations

The study has some limitations that could affect its findings. Firstly, participant selection might not represent the wider population since they were picked from specific communities. Secondly, participants’ responses may have been affected by desirability and recall biases—the information collected relied on what people said about themselves, which could be influenced by what they think is socially acceptable or by misunderstandings. The study only looked at what was happening at one point in time, so it might not show change over time. The study’s focus primarily on community perspectives related to MDA campaigns for NTDs may overlook other influential factors such as socioeconomic status and healthcare access, with uncontrolled external factors potentially influencing community perceptions and behaviours. Furthermore, certain demographic groups, like marginalized populations, may be underrepresented, and resource constraints could impact the study’s scope and ability to conduct follow-up studies.

## Figures and Tables

**Table 1:** Summary of data collection by method and by category.

Method	Number of Interviews	Category of Persons
Key Informant Interview	5	Community leaders
	4	Opinion leaders
	9	Community implementers (CDDs)
Focus Group Discussion	20	Adult Male (6) Adult Female (4) Youth Female (3) Male Youth (7)
Household Survey	1220	18–32 years; 33–47 years; 48–62 years; >62 years Female—644 (52.8%) Male—576 (47.2%)

**Table 2:** Awareness of NTD programmes in the two LGAs.

	Frequency (%)		
Awareness	Abaji	Bwari	Total
No	104 (15.1)	91 (17.1)	195 (16.0)
Yes	583 (84.9)	443 (82.9)	1.0 (84.0)
Total	687	533	1220
Reasons for unawareness			
No information	21 (20.2)	19 (21.2)	40 (20.5)
Unstable in the community	83 (79.8)	72 (78.8)	155 (79.5)
Total	104	91	195

**Table 3:** Respondents’ participation in NTD MDA campaigns.

	Proportion (%)		
Participation	Abaji	Bwari	Total
None	104 (15%)	3 (1%)	107 (9%)
Oncho/Lf	101 (14.7%)	343 (64%)	444 (36%)
Schisto/STH	482 (70.2%)	187 (35%)	669 (55%)
Total	687	533	1220

**Table 4:** Respondents’ engagement in the NTD MDA campaigns.

	Proportion (%)		
Engagement	Abaji	Bwari	Total
Attend community meetings regarding programme implementation	2.3	2.8	2.5
Distribute the drug	1.9	1.7	3.3
Nothing	3.5	7.3	5.2
Provide Health Education	1.9	1.3	1.6
Swallow drugs	84.7	83.1	82.5
Tell others about drug availability	5.7	3.8	4.84
Total	687	533	1220

**Table 5:** Willingness to participate in future MDA campaigns.

	Proportion (%)		
Participation	Abaji	Bwari	Total
No	1.0	2.1	1.5
Yes	99.0	97.9	98.5
**Total**	**687**	**533**	**1220**

**Table 6:** Perceived benefits of NTD drugs.

Benefits	Mectizan/Albendazole			Praziquantel		
	Frequency (%)			Frequency (%)		
	Abaji	Bwari	Total	Abaji	Bwari	Total
I feel better	526 (76.6)	414 (77.7)	**955 (78.3)**	541 (78.7)	166 (31.1)	**707 (58.0)**
No improvement in my health	7 (1.0)	6 (1.1)	**11 (0.9)**	5 (0.7)	3 (0.6)	**8 (0.6)**
Improvement but with adverse effects	50 (7.3)	22 (4.1)	**47 (3.9)**	25 (3.6)	14 (2.6)	**39 (3.2)**
**Total**	583	442	**1013**	571	183	**754**

**Table 7:** Performance rating and willingness to participate in future MDA.

Rating			
Frequency (%)			
Oncho/Lf	Abaji	Bwari	Total
Don’t know	259 (37.7)	101 (18.9)	359 (29.5)
Excellent	344 (50.1)	291 (54.6)	635 (52.1)
Poor	0	6 (1.1)	6 (0.5)
Moderate	84 (12.2)	135 (25.3)	220 (18.0)
**Total**	**687**	**533**	**1220**
**Schisto/STH**	**Abaji**	**Bwari**	**Total**
Don’t know	107 (15.6)	103 (19.3)	362 (29.7)
Excellent	484 (70.5)	291 (54.6)	635 (52.1)
Poor	0(0)	126 (23.6)	13 (1.07)
Moderate	96 (14.0)	13 (2.4)	210 (17.2)
	**687**	**533**	**1220**
**Inclination**			
**Frequency (%)**			
**Willingness**	**Abaji**	**Bwari**	**Total**
No	7 (1.0)	11 (2.1)	18 (1.5)
Yes	680 (99.0)	522 (97.9)	1202 (98.5)
**Total**	**687**	**533**	**1220**

In the context of the rating scale used in the study, “Don’t know” indicates respondents who were unsure or had no opinion about the performance of the NTD programmes, “Excellent” indicates a very positive perception of the programme’s performance, “Poor” indicates a very negative perception, and “Moderate” indicates a perception of average or moderate performance.

## Data Availability

All data is included in the manuscript, analysed data is not archived publicly due to privacy and would be made available on request.
